# Recent advances in materials and flexible electronics for peripheral nerve interfaces

**DOI:** 10.1186/s42234-018-0007-6

**Published:** 2018-05-23

**Authors:** Christopher J. Bettinger

**Affiliations:** 1 Department of Materials Science and Engineering, Carnegie Mellon University, 5000 Forbes Avenue, Pittsburgh, PA 15213 USA; 2 Department of Biomedical Engineering, Carnegie Mellon University, 5000 Forbes Avenue, Pittsburgh, PA 15213 USA

**Keywords:** Peripheral nerve interface, Biomaterials, Flexible electronics, Polymers

## Abstract

Peripheral nerve interfaces are a central technology in advancing bioelectronic medicines because these medical devices can record and modulate the activity of nerves that innervate visceral organs. Peripheral nerve interfaces that use electrical signals for recording or stimulation have advanced our collective understanding of the peripheral nervous system. Furthermore, devices such as cuff electrodes and multielectrode arrays of various form factors have been implanted in the peripheral nervous system of humans in several therapeutic contexts. Substantive advances have been made using devices composed of off-the-shelf commodity materials. However, there is also a demand for improved device performance including extended chronic reliability, enhanced biocompatibility, and increased bandwidth for recording and stimulation. These aspirational goals manifest as much needed improvements in device performance including: increasing mechanical compliance (reducing Young’s modulus and increasing extensibility); improving the barrier properties of encapsulation materials; reducing impedance and increasing the charge injection capacity of electrode materials; and increasing the spatial resolution of multielectrode arrays. These proposed improvements require new materials and novel microfabrication strategies. This mini-review highlights selected recent advances in flexible electronics for peripheral nerve interfaces. The foci of this mini-review include novel materials for flexible and stretchable substrates, non-conventional microfabrication techniques, strategies for improved device packaging, and materials to improve signal transduction across the tissue-electrode interface. Taken together, this article highlights challenges and opportunities in materials science and processing to improve the performance of peripheral nerve interfaces and advance bioelectronic medicine.

## Background

**Fig. 1:**
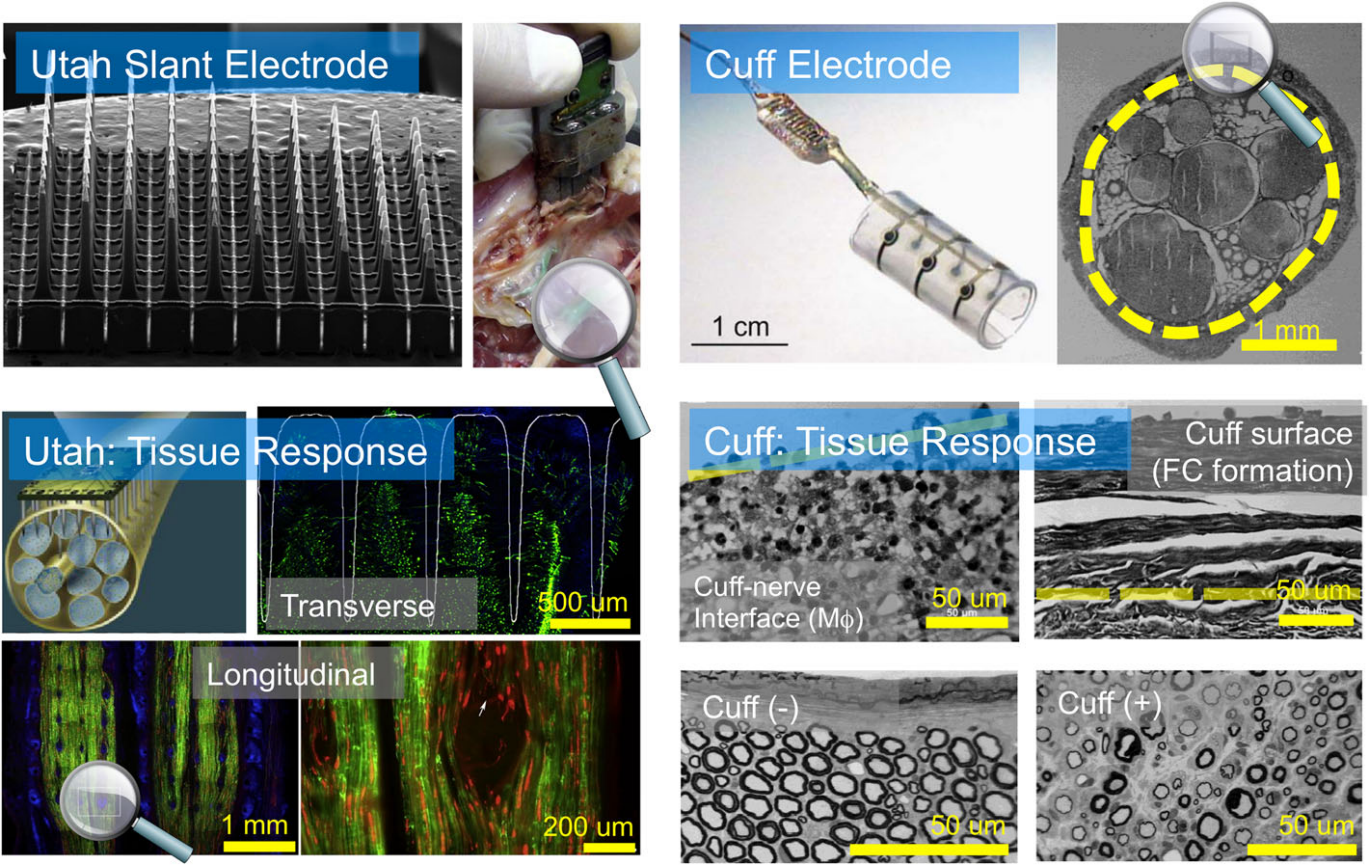
Peripheral nerve interfaces (PNI) consisting of both inserted probes and cuff electrodes damage the target tissue and alter the underlying physiology. Utah slant electrodes (left) inserted into feline sciatic nerves induces inflammation, glial scarring, and rearrangement of the fascicles as shown by histology. Retraction of nerve fibers (green) is shown in the transverse axis while rearrangement of the fibers is shown in the longitudinal axis. This figure is adapted from the following sources and used with permission: Grill et al. (copyright 2000 John Wiley & Sons) (Grill & Mortimer, 2000); Christensen et al. (copyright 2014, Springer) (Christensen et al., 2014)

Peripheral nerve interfaces (PNI) are implantable electronic medical devices that serve as a physical link between the natural nervous system and human-made computing environments. Implantable PNI are also invaluable tools for bioelectronic medicine because PNI can record and decode neural signals in the peripheral nervous system and provide an avenue for neuromodulation. Furthermore, implantable PNI can be surgically deployed with minimal invasiveness, yet can access many diverse therapeutic targets. PNI designed for electrical recording or stimulation of peripheral nerve targets often contain multielectrode arrays fabricated using materials commonly used in clinically approved medical devices for neuromodulation such as silicone-based substrates, platinum conductors, and polymer-based dielectrics. Multilectrode arrays are fashioned into cuffs that wrap nerve bundles or are embedded in microfabricated devices that are inserted into the tissue of the desired target. Microfabricated multielectrode arrays for PNI have grown in complexity leading to form factors that including Michigan arrays, Utah arrays, Utah slant arrays, transverse intrafascicular multielectrodes (TIME), longitudinal intrafascicular electrodes (LIFE), regenerative sieve electrodes, tissue-engineered electrodes, and other microscale multielectrode arrays (Grill et al., 2009). These devices often use off-the-shelf materials combined with unique design elements, exotic form factors, or non-conventional approaches to tissue integration (Spearman et al., 2017). Despite many novel designs for PNI in recent years, there remains a critical need to improve the overall performance and chronic reliability in vivo. PNI composed of cuff electrodes or inserted devices can damage local tissue. Penetration of the perineurium can cause tissue damage and a foreign body response while chronically implanted cuff electrodes can induce fibrosis and alter the fasciular structure of peripheral nerves (Fig. 1). The mechanical insult caused by PNI with any geometry can manifest as increased fibrous capsule formation, deterioration of fascicle structure, and a general perturbation of the underlying anatomy. Biological responses can compromise device performance, reduce chronic reliability, and lead to failure modes such as reduced signal-to-noise ratio during recording or increased stimulation thresholds during modulation (Nolta et al., 2015). Improving the in vivo performance of PNI will likely require the discovery, development, and successful deployment of novel materials.

**Fig. 2:**
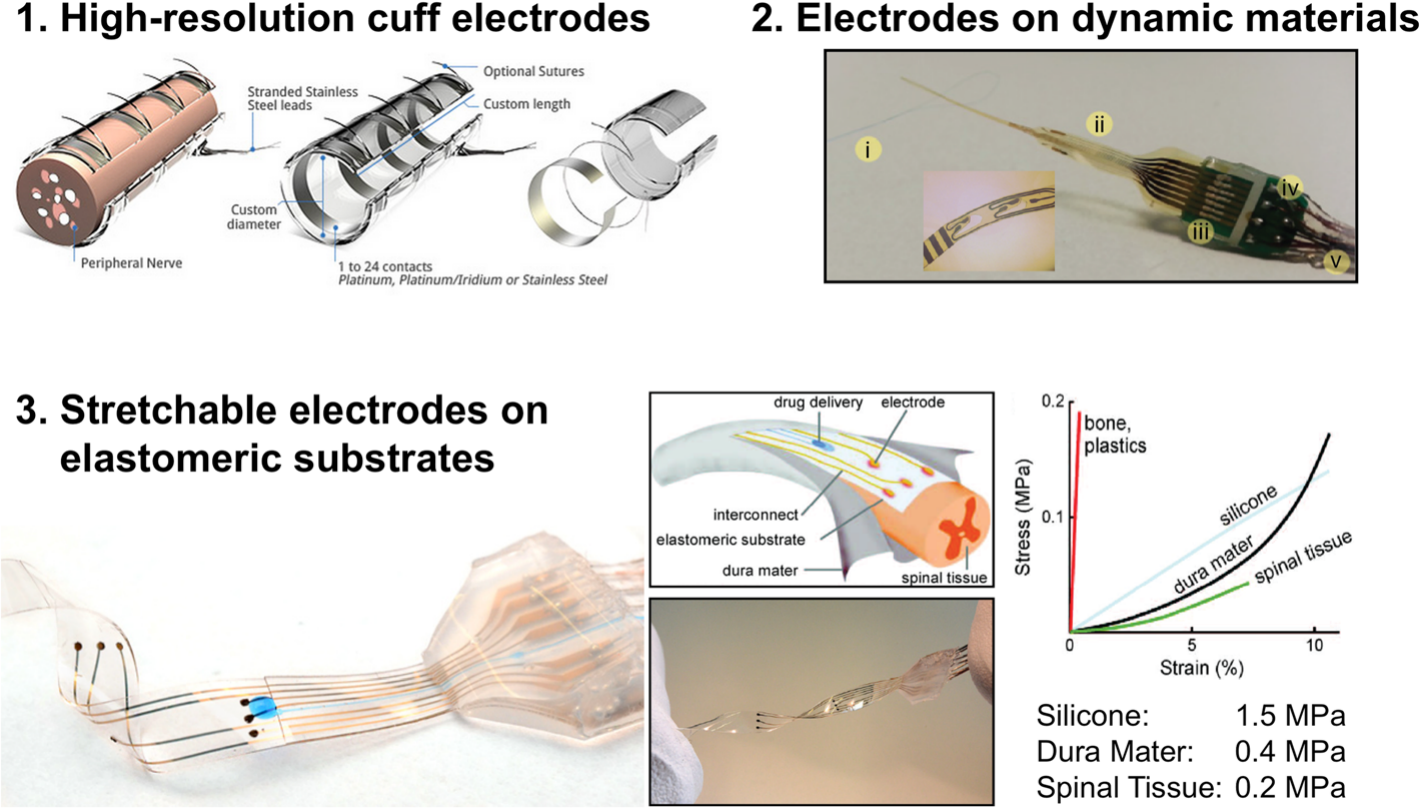
There are several important dimensions to consider in the design of peripheral nerve interfaces (PNI) with improved performance. 1) The electrode density can be improved through advances in device fabrication and manufacturing. The resolution of recording can also be improved by new form factors and materials that anchor the device in close proximity to the target tissue. 2) Electrodes can be fabricated on dynamic and smart materials that adopt unique shapes, respond to stimuli, or manage the in vivo tissue response. 3) PNI can be fabricated on flexible and stretchable substrates to match the mechanical properties of the target tissue. Elastomeric substrates such as silicones can match the properties of tissues in the spinal cord. Substrate materials with increased compliance will be required to match the mechanical properties of nerves in the PNS with smaller diameters. This figure is adapted from the following sources and used with permission: Cutrone et al. (copyright 2015, Institute of Physics)(Cutrone et al., 2015); Minev et al. (copyright 2015, American Association for the Advancement of Science) (Minev et al., 2015)

In addition to managing reliability at the tissue-device interface, there are several other dimensions of materials challenges that limit the performance, and therefore the practical application, of PNI as tools to advance bioelectronic medicine. Formidable materials challenges also include the following: reliable packaging materials and barrier layers for chronic reliability of flexible and stretchable electronic implants; novel electrode materials for abiotic-biotic signal transduction. This mini-review will highlight recent advances in novel materials, design, and microfabrication techniques for PNI (Fig. 2). Specially, this document will highlight materials challenges associated with advancing flexible PNI to improve mechanical matching at the tissue-device interface and novel packaging materials to increase the in vivo reliability of chronically implanted PNI. Lastly, novel materials and strategies to improve signal transduction across the abiotic-biotic interface are discussed. The technical content in this mini-review is intended to be introductory and by no means comprehensive. Also, there are numerous other topics that are integral to PNI performance that have been omitted due to space limitations. This article uses a materials scientist’s perspective to examine emerging trends in novel materials and device fabrication strategies with the goal of catalyzing new ideas in PNI design for use in bioelectronic medicine.

## Substrates and interconnects for flexible electrodes

### Polymeric substrates for flexible and stretchable electrodes

The current trend in PNI design aims to make these devices flexible, bendable, and stretchable to better interface with soft curvilinear tissues and reduce the mechanical mismatch at the abiotic-biotic interface. Polymer substrates such as polyimide and parylene-based polymers have enabled progress in flexible peripheral nerve interfaces in recent decades (Stieglitz et al., 2000; Stieglitz et al., 2005). Advances in flexible electronics are predicated on innovations in processing and micromachining of polymers (e.g. polyimides and parylenes) that can integrate microscale components and create form factors that are suitable for PNI (Noh et al., 2004a; Noh et al., 2004b; Ziegler et al., 2006). While flexible electronics permit conformal integration of electrodes with curvilinear sub-structures, stretchable electronics can accommodate strains in dynamic environments (Wagner & Bauer, 2012) where PNI are often implanted. Elastomers play a central role in advancing stretchable electronics for PNI. Silicone-based elastomers are attractive for implantable medical devices generally, and for stretchable electronics for neural recording more specifically. From a manufacturing standpoint, silicone-based elastomers such as poly(dimethylsiloxane) are cost-effective, robust, largely chemically inert in physiological environments, and amenable to established microfabrication techniques commonly used in PNI manufacturing such as replica-molding, transfer printing (Guo et al., 2013). Silicones offer desirable mechanical properties for bioelectronics including relatively low (and tunable) values for the Young’s modulus (*E*_PDMS_ ~ 10^5^ (Stieglitz et al., 2005)–10^6^ Pa), high extensibility, and bulk hydrophobicity (*γ*_PDMS_ = 19.8 mN/m; *γ*_PMMA_ = 41.1 mN/m) (Braley, 1970; Wu, 1971). From a regulatory perspective, silicones are ubiquitous medical materials with a rich history for use implants including PNI, specifically cuff electrodes for peripheral nerve recording and stimulation. Silicone-based devices are used in several clinically approved devices for neuromodulation including vagus nerve Silicones are highly stimulators (VNS Therapy System; Cyberonics, Houston, TX USA) (Espinosa et al., 1999; Schachter, 2002). Silicones are highly permeability to water vapor (*P*_H2O(v)-PDMS_ = 3600 × 10^− 9^ cm^3^(STP)-cm/s-cm^2^-cm Hg) (Spivack & Ferrante, 1969) compared to other barrier layers such as parylene-C (*P*_H2O(v)-Pary-C_ = 2 × 10^− 9^ cm^3^(STP)-cm/s-cm^2^-cm Hg)(Spivack & Ferrante, 1969; Charati & Stern, 1998; Favre et al., 1994; Robb, 1968). Despite this potential drawback, silicones continue to serve as the gold standard for substrates in PNI because flexible and stretchable interfaces can potentially exhibit improved biocompatibility compared to rigid probes.

### Bioabsorbable substrates and components for stretchable and bioresorbable electrodes

Bioabsorbale elastomers have utility as flexible and stretchable substrate materials for use in transient implantable electronic devices. Bioabsorbable PNI may be used in applications where recording or stimulation timelines may be relatively short – on the order of weeks or months. Bioabsorbable PNI are advantageous because they do not have to be explanted and therefore obviate many challenges associated with chronic implants. Towards this end, bioabsorbable elastomers are often composed of simple metabolizable monomers, hydrolytically active bonds, and hyperbranched amorphous networks to achieve biodegradability and extensibility. Prominent examples of bioabsorbable elastomers include polyurethane-ureas, poly(glycerol-*co*-sebacate) (PGS), poly(1,8-octanediol-*co*-citrate), polyurethanes, and their derivatives (Wang et al., 2002; Yang et al., 2004; Bettinger et al., 2008; Bettinger, 2011). Like silicones, bioabsorbable elastomers are extensible, can be synthesized in large quantities, processed into thin films, and are compatible with soft lithography and microfabrication techniques (Bettinger et al., 2006a; Bettinger et al., 2006b) including micromolding (Bettinger et al., 2006a), photocrosslinking (Nijst et al., 2007), and 3D-printing (Hung et al., 2014). Unlike silicones, bioabsorbable elastomers are often hygroscopic and susceptible to degradation through hydrolysis and enzymatic activity (Wang et al., 2003; Bettinger et al., 2009). Bioabsorbable elastomers offer utility as a substrate material for temporary implants that can eventually be resorbed within the body (Kim et al., 2010). Careful consideration must be given to the anticipated time scales of hydrolytic degradation versus desired device lifetime (Kim et al., 2010; Bettinger & Bao, 2010a; Bettinger & Bao, 2010b). Flexible and bioabsorbable substrates are of particular interest because they comprise the majority of the device and therefore determine the prospective toxicity risk of the implant. Much work has recently been pursued to design, engineer, and characterize various other active components for implantable electronics (Hwang et al., 2012; Kim et al., 2012). Active components include biocompatible organic semiconductors, silicon, pigments, biodegradable dielectrics, and metals that can be dissolved and metabolized such as magnesium (Irimia-Vladu, 2013; Hwang et al., 2014). These materials can be assembled into active and passive components such as transistors, capacitors, antennae, and other electronic components that may have utility in peripheral nerve interfaces (Chang et al., 2017). Many bioabsorbable devices have functional lifetimes on the order of weeks or months after implantation, which may be suitable for some acute applications, but less appropriate for situations where long-term stable function is required. Therefore, careful consideration must be granted to the final application of the implant.

### Ultracompliant materials for flexible electrode arrays

**Fig. 3:**
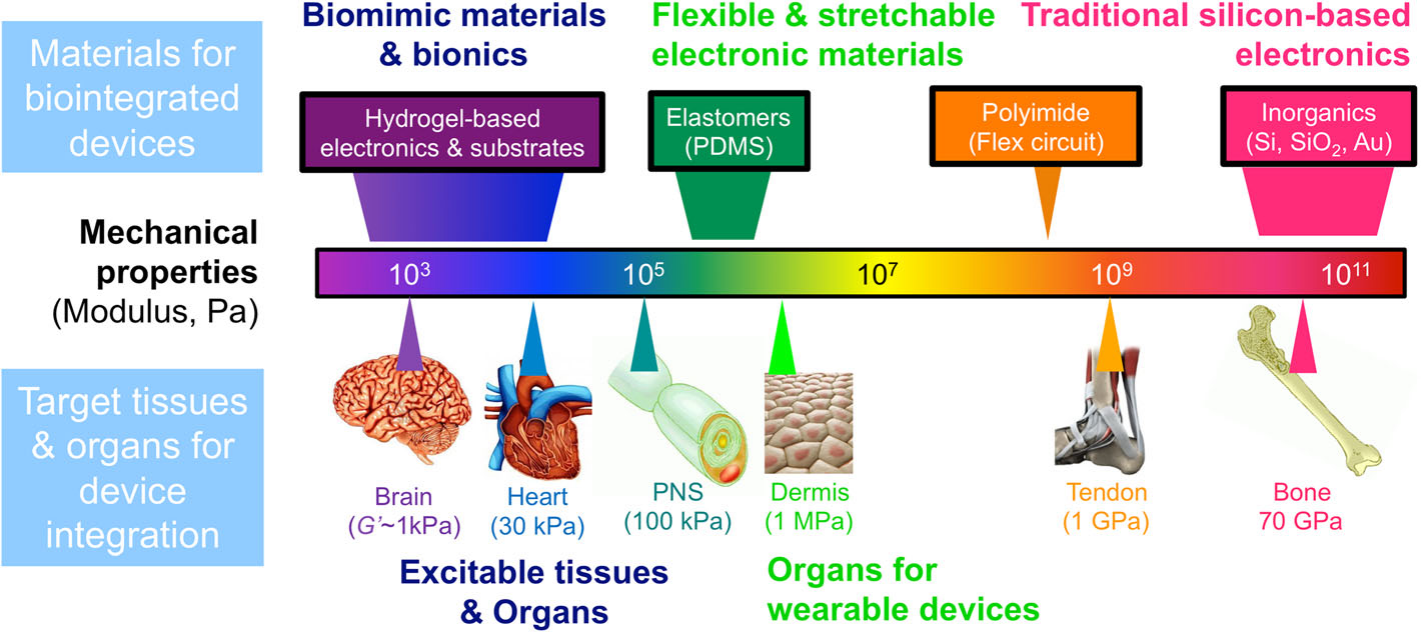
Modulus matching can guide the design of flexible and biointegrated electronic devices. The mechanical properties of various classes of materials used in microfabricated electronic devices (top) and organs in the human body (bottom) are plotted on a logarithmic spectrum of mechanical modulus. Materials traditionally used in microelectronic device fabrication (e.g. silicon, oxides, and metals) exhibit Young’s moduli that are orders of magnitude larger than the excitable tissues with which they are intended to interface including the peripheral nervous system. There are opportunities to engineer PNI with stretchable and ultracompliant electrode materials that can better match the mechanical properties of the target tissue

Improving the reliability of implantable bioelectronic devices including PNI remains an important directive that guides the design, evaluation, and adoption of new materials. Our current understanding of tissue-device interactions suggests that the performance and reliability of implantable devices can be improved by matching the mechanical properties at the biotic-abiotic interface. Biointegration can be improved, in part, by using flexible and stretchable electronic devices that match the mechanical properties of the target tissue (Minev et al., 2015). Peripheral nerves, cardiac muscle, and brain tissue exhibit elastic moduli as low as *G’*_brain_ ~ 1 kPa (Fallenstein et al., 1969), a value that is orders of magnitude smaller than most elastomers. Novel fabrication processes that integrate electronics with ultracompliant substrate materials, such as hydrogels, are therefore required for precise modulus matching at the tissue-PNI interface (Fig. 3). Hydrogel-based electronics have the potential to seamlessly interface with electronically excitable tissues in the peripheral nervous system (Wu et al., 2015).

### Extensible electronic components

Several engineering strategies have emerged to confer extensibility to electronic components that are integrated with flexible and stretchable substrate materials. One such strategy is to place active electronic components on mechanically rigid domains that are integrated in a continuous elastomeric domain (Lacour et al., 2006). Deterministic composites with differential Young’s moduli confine large strains to the flexible domains thereby reducing the effective strain on the active components. Metallic interconnects between active components must preserve their electrical conductivity while undergoing considerable tensile strains of > 100%. Non-deterministic composites can preserve electrical conductivity in high strain environments by employing mobile colloidal additives that maintain continuous contact during deformation (Kim et al., 2013; Ding et al., 2016). With respect to deterministic composites, there are several examples of compelling geometries to preserve the conductivity of microstructured metallic interconnects under large tensile strains. For example, serpentine structures can access out-of-plane deformation modes that reduce the intrinsic strain on any one microstructure (Zhang et al., 2013). The performance of serpentine interconnects can be enhanced by including secondary structures such as nested elements and fractal geometries. Other strategies for increasing the extensibility of conductors include fabricating 3D structures using Kirigami or Miura folding (Wang et al., 2017; Xu et al., 2017). Features that increase the extensibility of microfabricated conductors span multiple length scales, are largely complementary, and can be combined to preserve electronic properties under large strains.

## Non-conventional strategies for manufacturing and device fabrication

### Transfer printing of microstructures structures

Integrating functional electronics with polymer-based substrates represents a critical dimension in manufacturing flexible and biointegrated electronic devices for bioelectronic medicine. Recent innovations in soft materials processing and microfabrication can produce low-cost and flexible electronic devices. These techniques include advanced photolithography, ink-jet printing, and 3D-printing (Muth et al., 2014; Jakus et al., 2015). While useful for prototyping, many of these approaches present potential challenges in the scalable manufacturing of high-performance electronic devices such as high unit costs, poor reproducibility, and low yields (Macdonald et al., 2014). Transfer printing is a generalizable strategy for integrating electronic components with many substrate materials including elastomers, biodegradable polymers, protein matrices, and hydrogels. Transfer printing is an attractive approach to manufacture flexible and biointegrated electronics because the fabrication of the active components is fundamentally decoupled from the preparation of the substrate, a material that may not be compatible with processes that use elevated temperatures, vacuum, organic solvents, or corrosive buffers.

### Advances in transfer printing and heterogeneous device integration

The central technical challenge is transfer printing of any electronic device is achieving differential adhesion between the active layers and the various substrates. One way to dynamically control the adhesion between elastomeric substrates and thin stacks of active components is by altering the rate of lamination and delamination. This technique, termed kinetic control of adhesion, is used for heterogeneous integration of many inorganic active materials with elastomeric substrates in dry conditions in a scalable and reproducible way (Meitl et al., 2006). Other strategies for tunable adhesion between substrates and devices include altering surface chemistries (Sedó et al., 2013), dynamic control of texture (Wang & Xiao, 2017), or employing stimuli-responsive intermediate films. However, the novelty and relative complexity of many of these approaches prohibits widespread use in advanced manufacturing at present. Flexible and biointegrated electronics can benefit from ultracompliant hygroscopic materials with mechanical properties that match that of the target tissue. Technical challenges facing the heterogeneous integration of electronics with hydrated materials include poor adhesion in wet environments and delamination of components due to swelling and deswelling cycles. To this end, bioinspired materials design strategies enable the transfer printing of electronic structures to hydrogel-based substrates (Wu et al., 2015). Bioinspired adhesive motifs promote interfacial bonding between hydrogel substrates and many electronic materials such as noble metals, oxides, and polymers (Wu et al., 2016).

### 3D-printing and non-conventional fabrication

3D-printing affords another set of complementary strategies to integrate active electronics into soft materials. Recent achievements include the 3D-printing of conductive inks into shapes with complex topologies (Mannoor et al., 2013) to create biohybrid electronic devices. Flexible conductors with complex topologies can also be fabricated by infusing microstructures with liquid metals (Ladd et al., 2013; Gozen et al., 2014). Liquid metals afford many advantages over solid metallic counterparts including robust extensibility. Potential challenges in deploying liquid metals as stretchable conductors in implantable medical devices include preserving chemical stability in physiological conditions and managing toxicity risk.

## Advanced packaging materials for flexible electronics

Progress in device packaging, hermeticity, and on-board energy storage for PNI will likely emerge as a result of relentless, incremental innovation rather than fundamentally new approaches. Practical innovations in thin film processing, microfabrication, and materials integration will underpin many prospective technologies. Achieving long-term hermeticity in implantable medical electronics is a formidable challenge. This goal is especially challenging in devices that feature flexible and stretchable components. The ideal hermetic packaging would: be composed of a material that is stable in aqueous solutions; exhibit an extremely low liquid water; be insulating to electronic and ionic currents; use thin and conformal form factors that preserve flexibility and minimize the “dead space” between the encapsulation layers and the underlying components; maintain insulating and barrier properties under large strains.

### Trends in materials for flexible barrier layers

Chemical vapor deposition is a central materials processing strategy to create hermetic packaging. Conformal thin film barrier layers are desirable because films with submicron thicknesses preserve flexibility while conformal deposition reduces the “dead space” in packaged devices. Chemical vapor deposition is compatible with many reliable packaging materials including oxides, carbon-based materials, and polymers such as parylene and its derivatives. Thin parylene films are desirable packaging materials because they are hydrophobic barrier layers, present limited intrinsic biological activity, offer robust dielectric properties, and preserve mechanical flexibility (Loeb et al., 1977). Despite the relatively large hydraulic permeability of parylene compared to many inorganic films, flexible neural probes encapsulated in parylene-C achieve stable in vivo recordings for 12 months or longer (Hara et al., 2016). Nanoscale diamond is another emerging class of packaging materials (Narayan et al., 2011). Nanoscale diamond films have grain sizes that range from 2 to 5 nm (ultrananocrystalline) up to 100 nm (nanocrystalline diamond). Collectively, coatings composed of nanocrystalline diamond exhibit attractive mechanical and tribological characteristics coupled with excellent barrier and dielectric properties. Atomic layer deposition (ALD) is another versatile chemical vapor deposition technique to deposit films with molecular-scale precision. Thin film oxides deposited using ALD have gained traction as packaging materials for implanted medical electronics because the thickness and composition can be tightly controlled. One challenge is that oxides are susceptible to hydrolysis, albeit at very slow rates. Therefore, chronic implants packaged with some types of metal oxides must be co-encapsulated in other inert materials that limit water uptake into components. Flexible bilayers of nanometer-scale Al_2_O_3_ films and micron-scale parylene-C can greatly extend the in vitro stability of neural probes compared to devices encapsulated with parylene alone (Xie et al., 2012; Xie et al., 2014a).

### Bilayer and composite strategies for improving the performance of barrier layers

Maintaining impermeable barrier layers under high extensibility is challenging because even small strains can produce critical size defects, propagate cracks, and delaminate films. Inventing new techniques to process novel materials into robust barrier layers will likely require an amalgam of strategies from different disciplines. For example, barrier layers composed of bilayer structures with alternating layers of metal oxides and noble metals can dramatically improve hermeticity. Thin films of noble metals prevent water uptake and eliminate hydrolysis while thin films of oxides serve as a robust dielectric layer. The bilayer approach to improved hermeticiy extends to bilayers of Al_2_O_3_ and parylene-C (Xie et al., 2014b; Caldwell et al., 2017). Film delamination represents another critical mode for loss of hermeticity. Insulated electrodes with exposed windows create metal-insulator interfaces that are susceptible to failure and film delamination. Adhesion-promoting strategies are employed to address this issue. However, many bonding strategies are susceptible to the milieu of corrosive and reactive species in the biological environment such as salts, free radicals, and aqueous solutions with non-neutral pH values. Microwave heating of parylene-based encapsulation layers can also support robust bonding of polymer films between inorganic substrates (Noh et al., 2004b).

### Future perspectives on device packaging

There are many opportunities to advance the performance of materials and processing techniques for packaging, some of which are described here. Choosing the optimal packaging strategy for implantable devices to administer bioelectronic medicine depends on many factors including the implantation site, desired lifetime, anticipated modes of mechanical deformation, and performance requirements associated with barrier and dielectric properties. Polymers will continue to play a central role in packaging materials for implantable stretchable electronic devices. However, novel materials with improved dielectric properties, reduced permeability, and facile processing over existing commodity polymers will likely be required (Hassler et al., 2011). One particular concern is the performance of the barrier layer under tensile or compressive strain. Substantial strains can generate cracks internal stresses that can lead to delamination. Strategies for engineering stretchable metallic interconnects may be repurposed for the design and processing of packaging materials. Namely, techniques to increase the extensibility of materials with intrinsically high Young’s moduli such as serpentine networks, microstructures with out-of-plane geometries, and geometries that support affine deformations are also widely applicable to packaging materials including metals, oxides, and their composites. For example, one may consider fabricating barrier layers with topographically rich features where out-of-plane structures accommodate stretching and bending without reducing the barrier properties of the material (Wu et al., 2013; Münzenrieder et al., 2015).

## Electrode materials for bioelectronic signal transduction

**Fig. 4:**
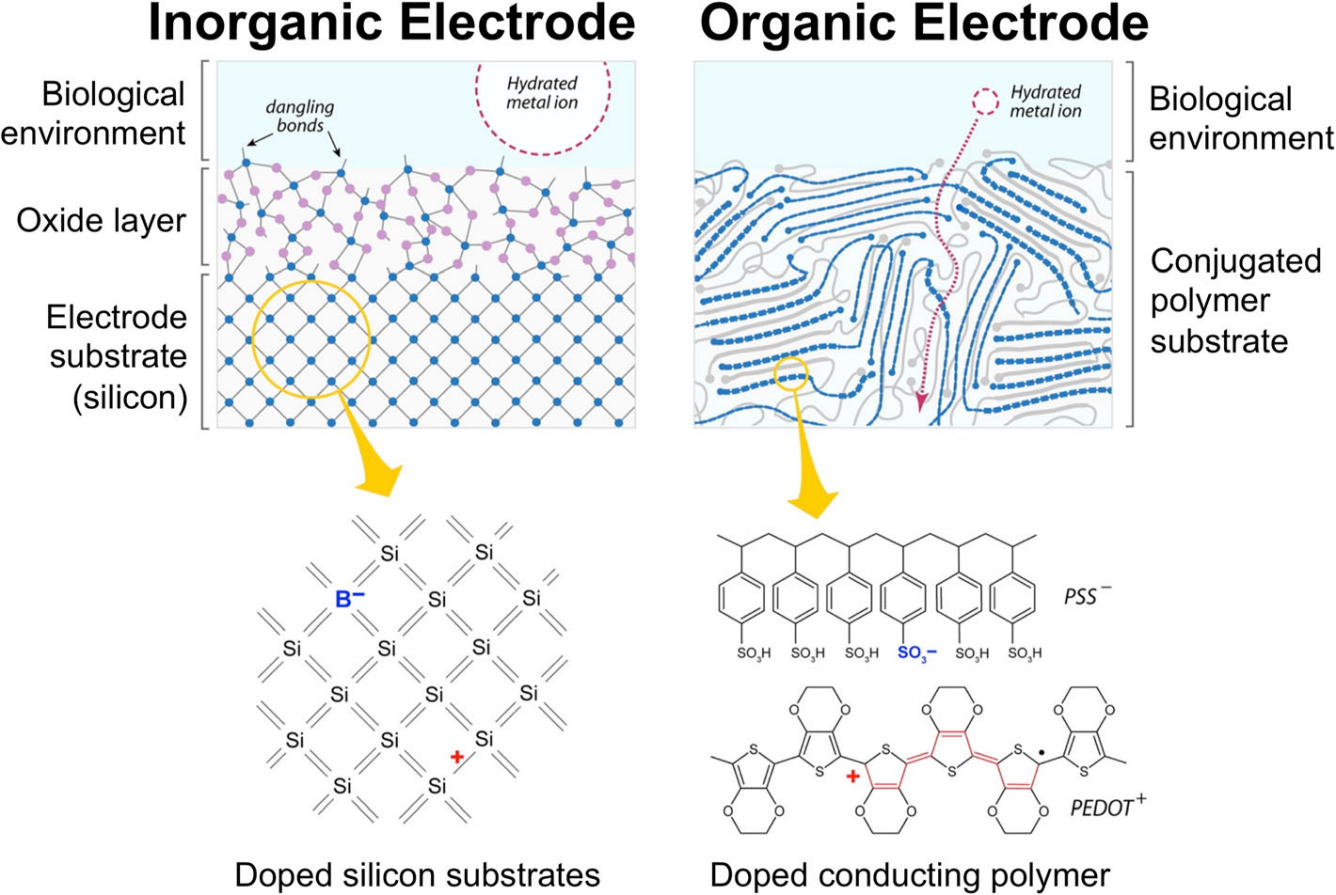
Peripheral nerve interfaces (PNI) that use organic electrodes offer at least two prominent advantages compared to interfaces composed of traditional inorganic materials. Silicon-based substrates are depicted here as an example. First, organic electrodes obviate the formation of oxide layers, which facilitates charge transport thereby potentially reducing the size of the electrode and improving spatial resolution. Second, organic electrodes, such as those composed of conducting poly(3,4-ethylenedioxythiophene) (PEDOT) doped with poly(styrene sulfonate) (PSS), can support both electronic and ionic transport. Organic electrodes therefore have the potential to transduce ionic signals emanating from neurons into electronic signals, which can be recorded by microelectronic devices (or vice versa). This figure is adapted from the following source and used with permission: Rivnay et al. (copyright 2014, American Chemical Society) (Rivnay et al., 2014)

Increasing the information bandwidth between the target tissue and the implanted device is critical for many applications for PNI in bioelectronic medicine. Bandwidth can be improved by increasing the electrode density in multielectrode arrays (Hoon Lee et al., 2016). Insertional PNI with 25 electrodes/mm^2^ have been used in various animal studies (Wark et al., 2013) while pilot studies of cuff-type electrodes in humans employed electrodes on the order of ~ 1 electrode/mm^2^ (Tan et al., 2015). While the ideal electrode resolution is highly context- and application-dependent, the fundamental limit on electrode size is ultimately governed by reliable signal transduction across the biotic-abiotic interface. Towards this end, a fundamental challenges to improving the spatial resolution of electrodes for implantable electronic devices (including PNI) that interface with the nervous system is to address the fundamental mismatch in the information currency; electrons in most human-made logic devices and ions in living cells and organisms. Electrodes prepared from inorganic materials such as Pt and TiN are largely chemically inert in aqueous environments and modest voltages (< 5 V) and thus employ capacitive coupling for both recording and stimulation. However, capacitive coupling using platinum (Pt) and titanium nitride (TiN) electrodes face limits on charge injection, which restricts the lower bound on electrode size (Weiland et al., 2002). Charge injection capacities (*CIC*) of ~ 1 mC/cm^2^ or larger can achieved by texturing electrodes to increase the geometric surface area (Cogan, 2008). However, novel electrode materials that transduce signals through electronic-ionic coupling can increase charge injection capacities and thus further miniaturize electrodes. Electrodes composed of redox-active inorganics such as activated iridium oxide (Cogan et al., 2004) (AIROF) use Faradaic charge transfer to increase the intrinsic injection capacity (*CIC*_AIROF_ ~ 1–4 mC/cm^2^). In addition to AIROF, iridium can also be deposited by DC reactive sputtering (SIROF), which can achieve comparable injection capacities to AIROF (Cogan et al., 2009). Iridium can also be alloyed alloyed with Pt to produce hybrid PtIr electrodes, although PtIr electrodes achieve in vitro *CIC* values several times smaller than AIROF (Cogan et al., 2005). Redox active conjugated polymers such as polypyrrole (PPy) and poly(3,4-ethylenedioxythiophene) (PEDOT) exhibit hybrid electronic-ionic conduction and can inject charge through Faradaic reactions (Green et al., 2013). PEDOT-modified electrodes can achieve charge injection capacities that approach values of *CIC*_PEDOT_ ~ 15 mC/cm^2^, a figure-of-merit that can reduce the characteristic dimension of stimulation electrodes by almost 4X compared to unmodified counterparts. Electrode stacks that use organic interfaces composed of conjugated polymers, for example, offer advantages over many inorganic electrodes such as the omission of oxide layers, (potentially) more efficient charge transport across the electrode, and efficient hybrid electronic-ionic transport (Fig. 4). Structure-property-processing relationships in conducting polymers are actively being explored to identify new dopants, novel geometries to increase the effective surface area, and elucidate novel charge transport mechanisms to improve signal transduction across the biotic-abiotic interface. The promise of high-performance organic coatings for electrodes coatings is tempered with practical challenges such as questionable long-term in vivo chemical stability and prospective regulatory challenges to qualify new materials.

## Conclusions, outlook, and opportunities for materials scientists

Improving the in vivo reliability and performance of chronically implanted PNI is critical to advancing scientific discovery and clinical translation of bioelectronic medicines. Most of the technical challenges that currently limit device performance are multifactorial, complex, and must be addressed by interdisciplinary efforts that span materials science, electrical engineering, mechanical engineering, and biomedical engineering. Many of these challenges arise from the fundamental asymmetries between the physical properties of the nervous system versus that of microfabricated electronic devices (Table 1). PNI reliability has steadily improved in recent decades, bolstered by novel materials processing strategies that have improved packaging, yield, and overall in vivo performance. Fundamental discoveries in polymer science have also expanded the materials toolbox available to engineers that design PNI. New technologies in flexible and biointegrated PNI will also be informed by expanding our collective knowledge of the underlying mechanisms that govern the foreign body response to implanted devices. New strategies to improve reliability may emerge including device miniaturization and bioactive components that either manage the underlying biological responses or more directly promote stable tissue-device integration. Understanding signal transduction at the tissue-device interface is another important dimension of device design that will be informed by fundamental studies of charge transport through biological structures, complex fluids, and functional materials such as conducting polymers. Discovering novel mechanisms for charge transport in soft matter or simply increasing our understanding of the governing principles could establish new paradigms for designing electrodes and interfaces for next-generation PNI.
